# Role of Dynamic 3 Tesla MRI in the Evaluation of Temporomandibular Joint Dysfunction

**DOI:** 10.7759/cureus.36681

**Published:** 2023-03-25

**Authors:** Pragya Surolia, Jitendra Kumawat, Pawan K Sharma

**Affiliations:** 1 Radiology, SMS (Sawai Man Singh) Medical College, Jaipur, IND; 2 Orthopedics, Geetanjali Medical College, Jaipur, IND

**Keywords:** mri, internal derangement, anterior disc displacement, temporomandibular joint dysfunction, temporomandibular joint

## Abstract

Introduction: The internal derangement of the temporomandibular joint (TMJ) is the most common type of dysfunction. Internal derangement can be divided into anterior and posterior disc displacement. Anterior disc displacement is the most common type, which is further classified into anterior disc displacement with reduction (ADDWR) and without reduction (ADDWoR). Temporomandibular joint dysfunction (TMD) symptoms are pain, reduced mouth opening, and joint sound. The main aim of this study was to correlate the clinical findings and magnetic resonance imaging (MRI) diagnosis of TMD in symptomatic and asymptomatic TMJs.

Methods: This prospective observational study was conducted in a tertiary care hospital on a 3T Philips Achieva MRI machine with 16-array channel coils after obtaining approval from the institutional ethical committee. A total of 60 TMJs of 30 patients were included in the study. After the clinical examination of each patient, an MRI of both right and left TMJs was done. In patients with unilateral TMD, the asymptomatic side was used as the asymptomatic joint, and the affected side as the symptomatic joint. Asymptomatic patients without any symptoms of TMD were used as controls for bilateral TMD cases. MRI with high-resolution specific serial sections was obtained in both open- and closed-mouth positions. A p-value of <0.05 was considered a statistically significant agreement between clinical and MRI diagnoses of internal derangement.

Results: Out of a total of 30 clinically asymptomatic TMJs, only 23 were normal on MRI. On MRI, 26 TMJs showed ADDWR and 11 showed ADDWoR. The most common shape of the disc was biconcave and the displacement was anterior in symptomatic joints. The most common type of articular eminence shape was sigmoid in ADDWR and flattened in ADDWoR. Agreement between clinical and MRI diagnosis in this study was 87.5% (p < 0.001).

Conclusion: The study concluded substantial agreement between clinical and MRI diagnosis of TMJ internal dysfunction and suggests that the diagnosis of the internal dysfunction can be made clinically but the exact position, shape, and type of disc displacement can be assessed precisely with the help of MRI.

## Introduction

The temporomandibular joint (TMJ) is a synovial joint formed by the mandibular condyle and the temporal bone [1,2]. The TMJ disc is a biconcave fibrous structure in between the articulating surface of the temporal bone and the mandibular condyle [2]. The disc and its attachments divide the joint into upper and lower compartments [3]. The most frequent cause of TMJ dysfunction is internal derangement [2]. The TMJ's internal derangement is defined as the abnormal position of the TMJ disc in relation to the mandibular condyle, articular eminence, and glenoid fossa. Trauma, bruxism, and physical and emotional stress can cause internal derangement of TMJ [4].

Internal derangement can be divided into anterior and posterior disc displacement. Anterior disc displacement is the most common type and can be subdivided into anterior disc displacement with reduction (ADDWR) and anterior disc displacement without reduction (ADDWoR) [5]. Conventional techniques like special radiographs (anterior-posterior, axiolateral oblique views), ultrasonography, arthroscopy, arthrography, and computed tomography are used for imaging of TMJ [2]. Magnetic resonance imaging (MRI) is the most accurate, non-invasive, non-ionizing imaging modality for the identification of the internal derangement of the TMJ. On MRI, disc position in both sagittal and coronal planes, dynamic evaluation of condylar translation, disc movement in open and closed mouth, disc morphology, joint effusion, synovitis, osseous erosions, and degenerative joint disease can be assessed. MRI is used to detect early changes of TMJ dysfunction to prevent the progression into the advanced and irreversible stage, which is characterized by condylar flattening and osteophytes [6].

## Materials and methods

Study design and inclusion/exclusion criteria

A prospective observational study was done in a tertiary care hospital. This study was approved by the institutional ethics committee of Gujarat MRI Center Pvt. Ltd. A total of 60 TMJs of 30 subjects of 20-50 years age group of both sexes were included. Subjects having pain in the TMJ or muscles of mastication and limitation in mouth opening were included. Asymptomatic subjects were also included in the study. Informed consent was obtained from the subjects. Subjects with a history of trauma to TMJ or previous surgery were excluded. All the subjects were evaluated for signs and symptoms of TMJ disorders including joint pain, restricted mouth opening, and joint sounds. Based on the clinical findings, all subjects were divided into symptomatic (Group I) and asymptomatic (Group II) TMJs.

Methodology

Each subject was explained the MRI procedure. In subjects with unilateral temporomandibular joint dysfunction (TMD), the asymptomatic side was used as the asymptomatic joint and the affected side as the symptomatic joint. Asymptomatic subjects without any symptoms of TMD act as controls for bilateral TMD cases. MRI was performed on 3T Philips Achieva MRI with 16-array channel coils and surface flex coil. The patient’s head was positioned with Frankfort's plane perpendicular to the MRI patient table. The axial localizer was set slightly in front of the external acoustic meatus through which oblique sagittal images were obtained perpendicular to the mandibular condyle. Oblique sagittal images were acquired in both closed mouth and open mouth. A plastic bite block was positioned between the incisor teeth for obtaining the maximum opening by stabilizing the mandible. MRI with high-resolution specific serial sections including T2 axial, T2 coronal, PD (proton density) sagittal oblique (open and closed mouth), and PD coronal oblique (open and closed mouth) were obtained. The position and shape of the articular disc, the shape of the articular eminence, and the presence of joint effusion were evaluated in both open- and closed-mouth positions.

**Figure 1:**
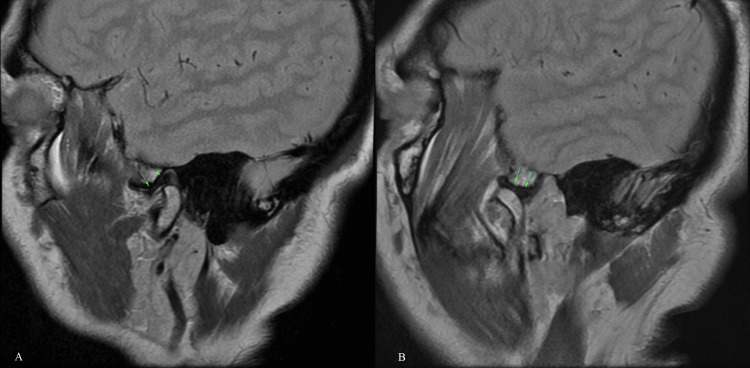
Normal TMJ disc in closed and open mouth Sagittal PD images: Normal closed mouth (A), the junction of the posterior band and bilaminar zone of disc is located superior/12 o'clock position of the condyle. Thin intermediate zone lies between the condyle and articular eminence (arrows). Normal open mouth (B), anterior translation of the condyle results in the condyle articulating with the inferior aspect of the articular eminence. The articular disc maintains its normal biconcave appearance (arrows). The intermediate zone of the articular disc is seen between the surfaces of the condyle and the eminence. TMJ, temporomandibular joint; PD, proton density.

Normal TMJ disc position is seen as a low-signal biconcave structure superior to the condyle above the apex of the condylar head in oblique sagittal images (Figure 1).

**Figure 2:**
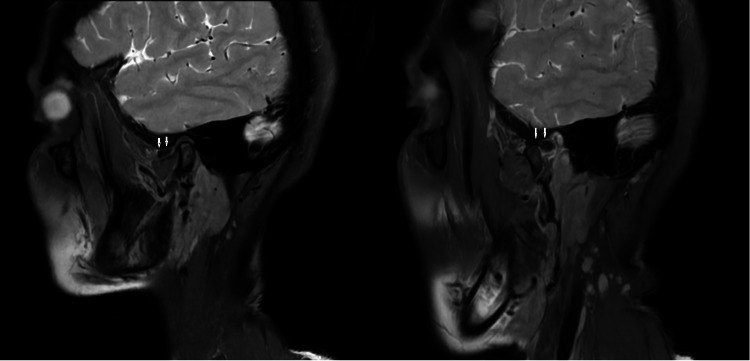
Anterior disc displacement with reduction Sagittal PD images: In the closed-mouth image, loss of biconcavity of the articular disc and anterior disc displacement (white arrow) are seen. In the open-mouth image, there is anterior movement of the condyle and the disc appears reduced (white arrow) from its abnormal position (ADDWR). PD, proton density; ADDWR, anterior disc displacement with reduction.

Disc displacement is seen as a low-signal disc and the high signal of the retro discal tissue located anterior to the superior aspect of the condyle in the sagittal plane. When the intermediate zone of the displaced disc during mouth closing is restored to the position between the articular eminence and articular surface of the condyle during mouth opening, it is called ADDWR (Figure 2).

**Figure 3:**
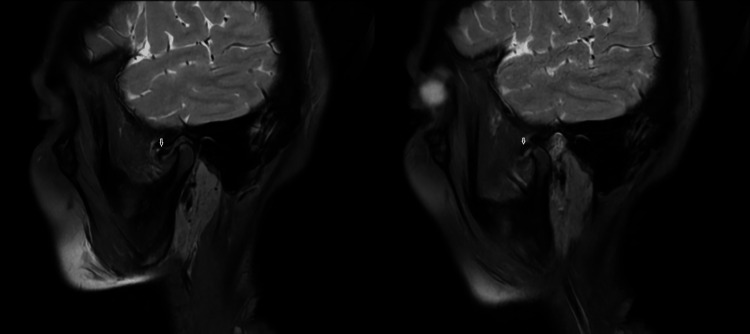
Anterior disc displacement without reduction Sagittal PD images: In the closed-mouth image, anterior displacement of articular disc is seen (white arrow). In the open-mouth image, the mandibular condyle is seen to glide anteriorly on the articular eminence. The disc remains displaced anterior to the mandibular condyle (white arrow) suggestive of anterior disc displacement without reduction. There is irregularity with mild crumpling of the articular disc seen. PD, proton density.

When the displaced disc is not restored to the normal position in the open mouth, it is called ADDWoR (Figure 3).

Statistical analysis

A chi-square test was done for analyzing categorical data. Kappa statistics were done to correlate the relationship between clinical and MRI diagnosis of TMJ's internal derangement. The k value between 0.6 and 0.8 was considered substantial agreement. A p-value <0.05 was considered significant.

## Results

A total of 60 TMJs of 30 subjects were included in the study. Out of the total 30 subjects, 22 were symptomatic subjects and eight were asymptomatic subjects. Out of 22 symptomatic subjects, there were eight males (36.3%) and 14 females (63.6%). Out of eight asymptomatic subjects, there were three (37.5%) males and five (62.5%) females. Out of 22 symptomatic subjects, 14 subjects had unilateral TMD and eight subjects had bilateral TMD, thus forming 30 symptomatic TMJs (Group I). The asymptomatic TMJs of the 14 subjects with unilateral TMD and the 16 TMJs of the eight asymptomatic subjects form 30 asymptomatic TMJs (Group II) (Table 1).

**Table 1:** Distribution of subjects Distribution of symptomatic and asymptomatic subjects by the presence of unilateral or bilateral displacement. TMD, temporomandibular joint dysfunction; TMJ, temporomandibular joint.

	Unilateral TMD	Bilateral TMD	Total
Symptomatic TMJ	14	8 x 2 (16)	30
Asymptomatic TMJ	14	8 x 2 (16)	30
Total	28	32	60

The mean and SD age of males and females in total in the study group was 29.36±4.59 years and 28.05±6.48 years, respectively. The mean and SD age of females and males in the symptomatic group was 29.00±6.93 years and 25.20±3.27 years, respectively. The mean and SD age of females and males in the asymptomatic group was 29.07±7.11 years and 25.38±3.50 years, respectively. The mean ages of females and males in both groups were almost similar with a p-value of 0.98 for femalfemales0.93 for males.

Out of the 60 TMJs, 37 (61.6%) showed disc displacement on MRI while 23 (38.3%) showed normally positioned discs. Among 30 symptomatic TMJs, two (6.6%) joints had normally positioned discs. Seventeen (56.6%) joints showed disc displacement with reduction and 11 (33.3%) joints had disc displacement without reduction. Out of a total of 30 asymptomatic joints, 21 (71%) joints had normal disc position and nine (30%) joints had disc displacement with reduction.

Among 60 TMJs, the most common position of disc displacement was the anterior displacement in 17 (28.3%) joints, followed by anterolateral in 11 (18.3%), lateral in 9 (15.0%), and 23 (38.3%) had normal disc position. Out of 17 anteriorly displaced discs, 13 (43.3%) were symptomatic while four (13.3%) were asymptomatic. There was a statistically significant correlation between anterior disc displacement and the presence of symptoms of TMD (p<0.001) (Table 2).

**Table 2:** Type of disc displacement Comparison of asymptomatic and symptomatic groups with type of disc displacement.

Type of disc displacement	Asymptomatic	%	Symptomatic	%	Total	%
Anterior	4	13.3	13*	43.3	17	28,3
Anterolateral	3	10.0	8	26.6	11	18.3
Lateral	2	6.6	7	23.3	9	15.0
Normal	21	70.0	2	6.6	23	38.3
Total	30		30		60	
	Chi-square= 25.5111; p = 0.00001*.(p-value<0.05)					

Among the total 60 discs, 49 (81.6%) had a biconcave shape in the closed-mouth position. In the open mouth position, the most common shape of the disc was biconcave in 42 (70%) out of 60 joints. The correlation between asymptomatic and symptomatic joints with the disc shape in the closed and open mouth was not statistically significant.

Among the total 60 joints, 44 (73.7%) had a sigmoid shape articular eminence, 12 (20%) joints had a flattened eminence, three (5%) had a box-shape eminence, and one (1.6%) had a deformed articular eminence. There was a statistically significant correlation between the type of disc displacement and the flattened shape of the articular eminence. The flattened shape of articular eminence was highly associated with anterior disc displacement without reduction (p<0.011).

Among the 60 joints, effusion was present in only 11 (18.33%) joints and was not observed in 49 (81.67%) joints. Among the 26 joints with ADDWR, joint effusion was observed in five (45.45%) joints whereas in the 11 joints with ADDWoR, six (54%) joints revealed joint effusion. However, joint effusion was also seen in two (18.18%) joints with no internal derangements. There was a statistically significant correlation between the presence of joint effusion and anterior disc displacement without reduction (p<0.0011).

Out of 60 TMJs, 30 (50%) joints were normal clinically whereas 23 (38.3%) joints were normal on MRI findings. Twenty-three (38.3%) joints were diagnosed as ADDWR clinically while 26 (43.33%) joints revealed ADDWR on MRI examination. In this study, seven (11.6%) joints were diagnosed as ADDWoR clinically as compared to 11 (18.3%) joints diagnosed as ADDWoR on MRI. From the kappa statistical analysis for comparison of clinical diagnosis and MRI diagnosis of internal derangement, the k-value was obtained as 0.6698, which reveals a substantial agreement between the two forms of diagnosis and was statistically significant (p<0.001) (Table 3).

**Table 3:** Clinical and MRI diagnosis *p<0.05. Agreement between clinical and MRI diagnosis is revealed (n=60). ADDWR, anterior disc displacement with reduction; ADDWoR, anterior disc displacement without reduction; MRI, magnetic resonance imaging.

Clinically	MRI diagnosis									
	Normal		ADDWR			ADDWoR		Total		%
Normal	21		9			0		30		50.0
ADDWR	2		17			4		23		38.3
ADDWoR	0		0			7		7		11.6
Total	23		26			11		60		
%	38.3		43.3			18.3				
Agreement		Expected agreement		Kappa	Std error		Z-value		p-Value	
87.5		62.1		0.6698	0.0981		6.8300		0.00001*	

## Discussion

Internal derangement of TMJ is best evaluated with MRI, as the articular disc can be directly visualized [7,8]. The MRI of the TMJ is used to evaluate the articular disc, its morphologic features, and its location relative to the condyle in both closed- and open-mouth positions [7].

In our study, out of 30 subjects, 22 were symptomatic subjects and eight were asymptomatic subjects. Out of 22 symptomatic subjects, there were eight males (36.36%) and 14 females (63.64%). So, there was a higher predilection of TMD in the female sex in our study, similar to the previously conducted studies [4,9-12].

A normal or superior position of the disc was seen in 38.3% (23) joints in this study. Whyte AM et al. [12] found normally positioned discs in 20% of the joints in their study. There was a statistically significant association between asymptomatic joints and superior disc position in previously conducted studies [9-11]. The prevalence of abnormal disc positions in the remaining TMJs showed anterior disc displacement to be the most commonly similar to the previous studies [9,10,12]. This observation suggests that disc displacement in the anterior direction is associated with symptomatic TMJ [9,10,13,14].

Out of the 37 displaced discs, 56.6% demonstrated disc reduction on mouth opening and 33.3% joints with disc displacement without reduction, in concordance with Whyte AM et al [12] where 59.5% of discs reduced and 40.5% of discs did not reduce on mouth opening. The clinical symptoms of these TMJs were pain and joint dysfunction.

We found the sigmoid shape of articular eminence to be the most common followed by the box shape and the flattened shape. The box shape of articular eminence was more commonly associated with ADDWR. The flattened shape of articular eminence was significantly more prevalent in joints with ADDWoR than with ADDWR. A flattened articular eminence was seen in 58.3% of joints with ADDWoR. This is in concordance with the previously conducted studies [14-16].

The results of this study correlate with those of the study conducted by Manfredini D et al. [17], which showed that the prevalence of joint effusion is higher in joints with ADDWoR (49.7%) compared to joints with ADDWR (24.7%). TMJ effusion can also be present in asymptomatic TMJs [17,18]. The prevalence rate of effusion in TMJs without pain has been 7% in a study done by Westesson PL and Brooks SL [18]. In this study, joint effusion was seen in 18.18% of the asymptomatic group.

Yatani H et al. [19] conducted a study on subjects with TMJ disorder to compare the clinical and MRI diagnosis; from the kappa statistical analysis of comparing the clinical and MRI diagnosis of internal derangement, the k-values were found to be 0.81 and 0.80, respectively. This was in concordance with the present study, which showed the k-value to be 0.6698 from the kappa statistical analysis for comparison of clinical diagnosis and MRI diagnosis of internal derangement, which reveals a substantial agreement and was statistically significant.

Limitation

Small sample size was a major limitation due to resource constraints. High cost, a long procedure time, and the need for the patient to have the mouth open for a longer duration were the limitations of this study.

## Conclusions

In conclusion, the present study found that disc displacement was more common in symptomatic TMJs compared to asymptomatic TMJs indicating that disc displacement causes symptoms of TMD. Disc displacement may be present in clinically asymptomatic TMJs. In this study, some asymptomatic TMJs showed internal derangement in the form of ADDWR which was diagnosed on MRI. Clinically there might be one joint with symptoms of TMD but disc displacement may be present on both sides, which can be diagnosed by MRI. In this study, biconcave-shape disc and anterior disc displacement of TMJ articular disc were the most commonly diagnosed.

This study concluded that there is a substantial agreement between the clinical and MRI diagnoses of TMJ internal dysfunction. It suggests that the diagnosis of internal dysfunction can be made clinically but the exact position, shape, and type of articular disc displacement can be assessed precisely with the help of MRI.
